# Obesity and Disease Severity Among Patients With COVID-19

**DOI:** 10.7759/cureus.13165

**Published:** 2021-02-05

**Authors:** Imane Motaib, Saad Zbiri, Saloua Elamari, Nezha Dini, Asma Chadli, Chafik El Kettani

**Affiliations:** 1 Department of Endocrinology, Diabetology, Metabolic Disease, and Nutrition, Mohammed VI University of Health Sciences (UM6SS), Casablanca, MAR; 2 Laboratory of Public Health, Health Economics and Health Management, International School of Public Health, Mohammed VI University of Health Sciences (UM6SS), Casablanca, MAR; 3 Department of Paediatrics, The International University Hospital Cheikh Khalifa Ibn Zaid, Casablanca, MAR; 4 Department of Paediatrics, Faculty of Medicine and Pharmacy, Mohammed V University of Rabat, Rabat, MAR; 5 Department of Anesthesia and Critical Care, Cheikh Khalifa International University Hospital, Mohammed VI University of Health Sciences (UM6SS), Casablanca, MAR

**Keywords:** obesity, covid-19, severity, sars-cov-2

## Abstract

Background

Obesity can be associated with one or more co-morbidities that worsen the effect of severe acute respiratory syndrome coronavirus 2 (SARS-CoV-2). Studies demonstrated that severe forms of coronavirus disease (COVID-19) have occurred in elderly patients and patients with co-morbidities such as diabetes, hypertension, and cardiovascular diseases.

Objective

This study investigated the impact of obesity on COVID-19 severity, irrespective of other individual factors.

Methods

This retrospective observational study included all adult patients with confirmed COVID-19 infection, who were admitted to Sheikh Khalifa Ibn Zaid International University Hospital between March 20 and May 10, 2020. First, we compared patients with and those without obesity in terms of demographic characteristics, co-morbidities, clinical symptoms, and outcomes. Further, using logistic regression models, we analyzed the association between obesity and intensive care unit (ICU) admission. Also, we examined whether the association between obesity and ICU admission was also consistent among overweight patients.

Results

The study population included 107 patients with confirmed COVID-19 infection. Obese patients have been admitted in ICU more than patients without obesity (P-value = 0.035). While adjusting for other risk factors for ICU admission, we found that obesity was an independent risk factor for ICU admission (OR = 5.04, 95% CI (1.14-22.37)). When we examined the association of both obesity and overweight with ICU admission, we found that only obesity was significantly associated with ICU admission (OR = 9.11, 95% CI (1.49-55.84)).

Conclusion

Our study found that obesity was strongly associated with severity of COVID-19. The risk of ICU admission is greater in the presence of obesity. Physicians should be awarded to the need of specific and early management of obese patients with COVID-19 disease.

## Introduction

Since December 2019, a new coronavirus has been identified in Wuhan and was named severe acute respiratory syndrome coronavirus 2 (SARS-CoV-2) [1]. The 2019 coronavirus disease (COVID-19) has spread rapidly around the world, and the World Health Organization (WHO) has declared on March 11, 2020, the infection as a global pandemic [2]. According to the World Health Organization (WHO) data, more than five million of the population have been infected and over 300,000 deaths have been reported worldwide [3]. Symptoms of COVID-19 disease range from mild flu-like symptoms to serious respiratory illness such as acute respiratory distress syndrome (ARDS) [4].

Obesity is a major public health problem that is characterised as a pandemic. It can be associated with one or more co-morbidities that worsen the effect of SARS-CoV-2 [1]. Indeed, studies demonstrated that severe forms of COVID-19 have occurred in elderly patients and patients with co-morbidities such as diabetes, hypertension, and cardiovascular diseases [1,5]. In addition, obesity can play an important role in the evolution of COVID-19 infection; severe obesity is associated with several respiratory problems including sleep apnea syndrome, surfactant dysfunction, and restrictive lung disease [6]. Furthermore, obese patients pose a serious challenge especially for intubation and nursing. Also, it has been demonstrated that obesity is an independent risk factor for hospitalization and death in other respiratory infections such as the H1N1 flu [7].

Despite a preponderance of evidence that obesity is associated with poor COVID-19 outcomes, very few data have been published about the impact of obesity on population infected with SARS-CoV-2.

The present study investigated the impact of obesity on patients admitted for COVID-19 to Sheikh Khalifa Ibn Zaid International University Hospital, in Casablanca, Morocco. Our hypothesis was that patients with obesity are more likely to present a severe form of COVID-19.

## Materials and methods

Study design and population

This retrospective observational study evaluated patients hospitalized with confirmed COVID-19 infection, based on the World Health Organization interim guidance [8]. We included all adult patients with laboratory confirmed SARS-Cov-2 infection, using a reverse transcriptase-polymerase chain reaction assay, who were admitted to Sheikh Khalifa Ibn Zaid International University Hospital between March 20 and May 10, 2020. This hospital has been mandated by the Moroccan Ministry of Health to take care of patients with COVID-19. Depending on their severity of COVID-19, patients were admitted to the intensive care unit (ICU) (severe patients) or not (non-severe patients). We excluded from our study pregnant women and those patients under the age of 18.

Data collection

Data extraction was performed by a trained team of physicians from electronic medical records of patients. Investigators collected and reviewed patient data. We extracted demographic characteristics (age, gender), anthropometric measures (weight and height), co-morbidities (hypertension, diabetes, cardiovascular disease, respiratory disease, dyslipidemia, and other diseases such as hyperuricemia, neoplasia, or smoking), clinical symptoms (general symptom including fever, respiratory symptom, ear, nose and throat (ENT) symptom, and digestive symptom) and clinical outcomes (ICU admission, use of invasive ventilation, onset of complications (acute respiratory distress syndrome (ARDS), secondary infection, multiple organ failure, thromboembolic complication), and death.

Outcomes assessment

According to the WHO classification, obesity was defined as having a body mass index (BMI) greater than or equal to 30 kg/m² (BMI ≥ 30 kg/m²), overweight as a BMI from 25 kg/m² to 29.9 kg/m² (25 kg/m² ≤ BMI < 30 kg/m²) and normal weight as a BMI from 18.5 kg/m² to 24.9 kg/m² (18.5 kg/m² ≤ BMI < 25 kg/m²) [9]. The lowest BMI was equal to 18.5.

First, we compared patients with and those without obesity in terms of demographics characteristics, co-morbidities, clinical symptoms, and outcomes. Second, we analyzed the association between obesity and ICU admission. Third, we examined whether the association between obesity and ICU admission was also consistent among overweight patients.

Severity of COVID-19 was based on ICU admission. Criteria of ICU admission followed the WHO interim guidance [8].

Statistical analysis

For the descriptive analysis, we represented continuous measurements as medians and interquartile ranges (IQRs) and we compared them using nonparametric k-sample test on the equality of medians. Categorical data were described as percentages and frequencies and were compared using the Fisher exact test. Secondly, univariate analysis was performed for all variables. Multivariate logistic regression was implemented to examine the association between ICU admission and all significant variables in the univariate analysis. Results were reported as odds ratios (ORs) and 95% confidence intervals (CIs). To establish the robustness of dataset and analysis, we also performed a stepwise multivariate analysis based on a bidirectional elimination in order to take into account the possible correlation that may exist between some variables particularly those of comorbidities. Statistical analyses were performed using STATA software. All P-values were two-sided, and those < 0.05 were considered statistically significant.

Ethics

The study was approved by the institutional ethics board of Sheikh Khalifa Ibn Zaid International University Hospital (approval number: CE_UM6SS/1/06/2020 - April 3, 2020). Due to the retrospective type of the study, no patient consent was required as the study did include only unidentified data, in accordance with the national law.

## Results

The study population included 107 hospitalized patients with confirmed COVID-19 infection. Characteristics of the study population are presented in Table 1. The median age of patients was 53 years old with an interquartile between 36 and 64 years old. Of these patients, 59.8% were men. The prevalence of obesity was 22.4% while 38.3% of patients had an overweight, and 39.3% had a normal weight. Most common co-morbidities were hypertension (30.8%), diabetes (15%), and cardiovascular disease (15%). The most prevalent clinical symptoms were respiratory symptoms (63.6%) and fever (49.5%). The most common complication was secondary infection (21.5%). We noted that 39.3% of patients were admitted to ICU and 12.2% died.

**Table 1:** Characteristics of the study population. BMI: body mass index; ENT: ear, nose and throat; ARDS: acute respiratory distress syndrome; ICU: intensive care unit.

	Median (IQR) or N (%)
Demographics	
Age, years	53 (36-64)
Male	64 (59.8)
BMI, kg/m^2^	
Normal (<25)	42 (39.3)
Overweight (25-29.9)	41 (38.3)
Obesity (≥ 30)	24 (22.4)
Comorbidities	
Hypertension	33 (30.8)
Diabetes	16 (15)
Cardiovascular disease	16 (15)
Respiratory disease	9 (8.4)
Dyslipidemia	10 (9.4)
Other disease	13 (12.2)
Clinical symptoms	
Fever	53 (49.5)
General symptom	45 (42.1)
Respiratory symptom	68 (63.6)
ENT symptom	32 (29.9)
Digestive symptom	26 (24.3)
Outcomes	
Secondary infection	23 (21.5)
Invasive mechanical ventilation	13 (12.3)
ARDS	13 (12.3)
Thromboembolic complication	4 (3.7)
Multi-organ failure	9 (8.4)
ICU admission	42 (39.3)
Death	13 (12.2)

The characteristics of the study population according to their BMI status are reported in Table 2. Compared to non-obese patients, patients with obesity were significantly older (61 years old versus 49 years old, P = 0.012). Obese patients were also more likely to have hypertension than those without obesity (62.5% versus 21.7 %, P < 0.001). In terms of clinical symptoms, patients with obesity had less digestive symptoms than patients without obesity (P = 0.007). Otherwise, these patients were comparable in terms of other clinical symptoms. Regarding clinical outcomes, there was no significant difference between obese and non-obese patients for complications including secondary infection, ARDS, multi-organ failure, and thromboembolic complication. The use of mechanical ventilation was also comparable, regardless of the patient obesity status. However, obese patients had an ICU admission rate significantly higher than the rate of non-obese patients (58.3% versus 33.7%, P = 0.035). Finally, 25% of obese patients died while 8.4% of non-obese patients died (P = 0.069).

**Table 2:** Characteristics of the study population according to their obesity status. BMI: body mass index; IQR: interquartile range; ENT: ear, nose and throat; ARDS: acute respiratory distress syndrome; ICU: intensive care unit.

	Non-obese (BMI < 30 kg/m^2^), N = 83	Obese (BMI ≥ 30 kg/m^2^), N = 24	P-value
Demographics			
Age, years, median (IQR)	49 (34-63)	61 (49-68.5)	0.012
Male, N (%)	51 (61.5)	13 (54.2)	0.637
Comorbidities			
Hypertension, N (%)	18 (21.7)	15 (62.5)	0.000
Diabetes, N (%)	10 (12.1)	6 (25)	0.189
Cardiovascular disease, N (%)	12 (14.5)	4 (16.7)	0.753
Respiratory disease, N (%)	7 (8.4)	2 (8.3)	1.000
Dyslipidemia, N (%)	7 (8.4)	3 (12.5)	0.690
Other disease, N (%)	11 (13.3)	2 (8.3)	0.728
Clinical symptoms			
Fever, N (%)	41 (49.4)	12 (50)	1.000
General symptom, N (%)	38 (45.8)	7(29.2)	0.166
Respiratory symptom, N (%)	52 (62.7)	16 (66.7)	0.812
ENT symptom, N (%)	25 (30.1)	7(29.2)	1.000
Digestive symptom, N (%)	25 (30.1)	1 (4.2)	0.007
Outcomes			
Secondary infection, N (%)	15 (18.1)	8 (33.3)	0.156
Invasive mechanical ventilation, N (%)	8 (9.6)	5 (20.8)	0.161
ARDS, N (%)	10 (12.1)	3 (12.5)	1.000
Thromboembolic complication, N (%)	4 (4.8)	0 (0)	0.573
Multi-organ failure, N (%)	6 (7.2)	3 (12.5)	0.416
ICU admission, N (%)	28(33.7)	14 (58.3)	0.035
Death, N (%)	7 (8.4)	6 (25)	0.069

As represented in Table 3, in the univariate analysis, obesity was associated with ICU admission (OR = 2.75, 95% CI (1.08-6.97)). Other variables, including age, gender, co-morbidities (diabetes, hypertension, cardiovascular disease, and other disease) and respiratory symptoms were also associated to ICU admission. We also performed a multivariate analysis, and found that obesity was associated with ICU admission, independently of other variables (OR = 5.24, 95% CI (1.05-26.20)). This result was verified by a stepwise multivariate analysis model (OR = 5.04, 95% CI (1.14-22.37)).

**Table 3:** Association of obesity with ICU admission. ICU: intensive care unit; BMI: body mass index; ENT: ear, nose and throat.

	Univariate OR (95% CI)	Multivariate model OR (95% CI)	Multivariate model (stepwise) OR (95% CI)
Demographics			
Age, years	1.09 (1.06-1.13)	1.09 (1.04-1.15)	1.11 (1.06-1.16)
Male	4.02 (1.66-9.73)	7.87 (1.67-37.15)	6.58 (1.57-27.48)
Obesity			
No (BMI < 30 kg/m^2^)	1	1	1
Yes (BMI ≥ 30 kg/m^2^)	2.75 (1.08-6.97)	5.24 (1.05-26.20)	5.04 (1.14-22.37)
Comorbidities			
Hypertension	5.40 (2.22-13.11)	1.03 (0.24-4.43)	
Diabetes	6.10 (1.81-20.52)	2.28 (0.43-12.22)	
Cardiovascular disease	9.26 (2.45-35.05)	2.46 (0.35-17.35)	
Respiratory disease	2.06 (0.52-8.16)		
Dyslipidemia	1.04 (0.27- 3.91)		
Other disease	4.16 (1.19- 14.54)	25.33 (2.79-230.19)	26.66 (3.12-227.54)
Clinical symptoms			
Fever	1.94 (0.88-4.27)		
General symptom	2.01 (0.91-4.43)		
Respiratory symptom	4.85 (1.88-12.49)	10.67 (2.45-46.53)	10.12 (2.49-41.20)
ENT symptom	1.09 (0.47-2.53)		
Digestive symptom	1.46 (0.60-3.56)		

As a secondary step, represented in Table 4, we examined the association between obesity, along with overweight, and ICU admission. We found that only obesity was significantly associated with ICU admission (OR = 9.55, 95% CI (1.36-67.29)). This result has also been confirmed by the stepwise multivariate analysis model (OR = 9.11, 95% CI (1.49-55.84)).

**Table 4:** Association of overweight and obesity with ICU admission. ICU: intensive care unit; BMI: body mass index; ENT: ear, nose and throat.

	Univariate OR (95% CI)	Multivariate model OR (95% CI)	Multivariate model (stepwise) OR (95% CI)
Demographics			
Age, years	1.09 (1.06-1.13)	1.10 (1.04-1.16)	1.11 (1.06-1.17)
Male	4.02 (1.66-9.73)	7.81 (1.59-38.27)	6.24 (1.45-26.78)
BMI, kg/m^2^			
Normal (< 25)	1	1	1
Overweight (25-29.9)	1.29 (0.52-3.20)	2.50 (0.56-11.21)	2.56 (0.60-11.05)
Obesity (≥ 30)	3.12 (1.10-8.86)	9.55 (1.36-67.29)	9.11 (1.49-55.84)
Comorbidities			
Hypertension	5.40 (2.22-13.11)	1.02 (0.24-4.43)	
Diabetes	6.10 (1.81-20.52)	2.02 (0.37-11.11)	
Cardiovascular disease	9.26 (2.45-35.05)	2.64 (0.35-19.71)	
Respiratory disease	2.06 (0.52-8.16)		
Dyslipidemia	1.04 (0.27-3.91)		
Other disease	4.16 (1.19- 14.54)	31.35 (3.12-314.53)	32.80 (3.51-306.56)
Clinical symptoms			
Fever	1.94 (0.88-4.27)		
General symptom	2.01 (0.91-4.43)		
Respiratory symptom	4.85 (1.88-12.49)	12.02 (2.65-54.52)	11.20 (2.67-47.05)
ENT symptom	1.09 (0.47-2.53)		
Digestive symptom	1.46 (0.60-3.56)		

## Discussion

Our study aimed to investigate the association of obesity with the severity of COVID-19. The prevalence of obesity in our study was 22.4%. This prevalence appears to be higher than the prevalence of obesity in the general population in Morocco, which is less than 20% [10]. Indeed, this higher prevalence of obesity among patients with COVID-19 has been reported in previous studies. In a cohort of 340 patients, authors found that the prevalence of obesity was 1.35 times higher in patients with severe COVID-19 than in the general French population [11]. This finding has also been demonstrated in obese patients with other viral respiratory infections including H1N1 influenza. In fact, during the H1N1 pandemic, hospitalization rates among obese patients were greater than patients without obesity [12].

Our study has also demonstrated that patients with obesity were older and had significantly a higher prevalence of hypertension than non-obese patients. No statistically significant differences between obese and non-obese patients were found in terms of clinical symptoms, complications and death. However, our study found that among obese patients, more than half of them were admitted to the intensive care unit.

Multivariate analysis showed that obesity was independently associated with severity of COVID-19. After adjustment for other variables including co-morbidities, odds of obesity were significantly higher in patients with severe COVID-19. Moreover, unlike obesity, being overweight was not associated with severity of COVID-19, compared to normal weight.

These findings are in agreement with previous reports which linked obesity with severity of COVID-19. In a French cohort of 124 patients infected by COVID-19, those patients with obesity represented 47.6% of patients admitted in ICU, and those with BMI ≥ 35 kg/m² were more likely to require invasive mechanical ventilation. In a similar study, authors showed that patients with moderate obesity were 1.8 times more likely to be admitted to critical care unit. Moreover, Kass et al. reported that obesity conferred greater severity in younger patients with COVID-19. They found a negative correlation between BMI and age among patients with COVID-19 admitted in ICU [13].

The exact causes explaining the link between obesity and the severity of COVID-19 are poorly known. However, several mechanisms may explain the increased risk of progression to severity in obese patients with COVID-19 infection. First, obesity impacts pulmonary function by affecting the ventilatory mechanics and inducing obese hypoventilation syndrome which contributes to respiratory failure [14,15]. Second, the chronic low-grade inflammation typically described in obesity may contribute to the onset of cytokine storm, which is a predictor of poor disease progression [16]. Also, the adipose tissue may represent a reservoir for viral spread because of the higher expression of Angiotensin conversion enzyme 2 (ACE 2) receptors, which are the sites of entry of SARS-CoV-2 [17,18]. Additionally, obesity is associated to a prothrombotic state which could precipitate thrombotic complications of COVID-19 [19]. Obesity is also associated with other co-morbidities such as diabetes, hypertension, and cardiovascular disease which represent an important factor for severe illness and death by COVID-19 [1,5]. 

Our study has some limitations. First, the study included limited number of patients. Second, due to the small sample of the study, we could not study all the different categories of obesity and their association with the COVID-19 severity. Third, this was a single-centre study, further multicentre and prospective studies should be conducted to validate our results.

However, our study has many strengths. Our data were collected by a trained team of physicians from electronic medical records of patients. Medical investigators collected and reviewed all patient data which made it of high quality. Further, we had access to different patient characteristics including demographics, co-morbidities, clinical symptoms, and outcomes, which made it possible to take into account all these factors in the multivariate analysis.

## Conclusions

Our study found that obesity was strongly associated with severity of COVID-19. The risk of ICU admission is greater in the presence of obesity. Physicians should be apprised of the need for specific and early management of obese patients with COVID-19 disease. Also, the barrier measures to prevent the infection by the COVID-19 virus should be drastically respected by patients with obesity. Finally, vaccination, whenever available, should be considered for patients with obesity.
